# Generation and transmission of interlineage recombinants in the SARS-CoV-2 pandemic

**DOI:** 10.1016/j.cell.2021.08.014

**Published:** 2021-09-30

**Authors:** Ben Jackson, Maciej F. Boni, Matthew J. Bull, Amy Colleran, Rachel M. Colquhoun, Alistair C. Darby, Sam Haldenby, Verity Hill, Anita Lucaci, John T. McCrone, Samuel M. Nicholls, Áine O’Toole, Nicole Pacchiarini, Radoslaw Poplawski, Emily Scher, Flora Todd, Hermione J. Webster, Mark Whitehead, Claudia Wierzbicki, The COVID-19 Genomics UK (COG-UK) Consortium, Nicholas J. Loman, Thomas R. Connor, David L. Robertson, Oliver G. Pybus, Andrew Rambaut

**Affiliations:** 1 Institute of Evolutionary Biology, University of Edinburgh, Edinburgh EH9 3FL, UK; 2 Center for Infectious Disease Dynamics, Department of Biology, Pennsylvania State University, University Park, PA 16802, USA; 3 Pathogen Genomics Unit, Public Health Wales NHS Trust, Cardiff CF14 4AY, UK; 4 Institute of Integrative Biology, University of Liverpool, Liverpool L69 7ZB, UK; 5 Institute of Microbiology and Infection, University of Birmingham, Birmingham B15 2TT, UK; 6 The COVID-19 Genomics UK (COG-UK) Consortium, https://www.cogconsortium.uk/; 7 School of Biosciences, Cardiff University, Cardiff CF10 3AX, UK; 8 MRC-University of Glasgow Centre for Virus Research (CVR), Glasgow G61 1QH, UK; 9 Department of Zoology, University of Oxford, Oxford OX1 3SZ, UK; 10 Department of Pathobiology and Population Sciences, The Royal Veterinary College, London AL9 7TA, UK

**Keywords:** SARS-CoV-2, genomics, evolution, recombination, genomic epidemiology, B.1.1.7, variants

## Abstract

We present evidence for multiple independent origins of recombinant SARS-CoV-2 viruses sampled from late 2020 and early 2021 in the United Kingdom. Their genomes carry single-nucleotide polymorphisms and deletions that are characteristic of the B.1.1.7 variant of concern but lack the full complement of lineage-defining mutations. Instead, the remainder of their genomes share contiguous genetic variation with non-B.1.1.7 viruses circulating in the same geographic area at the same time as the recombinants. In four instances, there was evidence for onward transmission of a recombinant-origin virus, including one transmission cluster of 45 sequenced cases over the course of 2 months. The inferred genomic locations of recombination breakpoints suggest that every community-transmitted recombinant virus inherited its spike region from a B.1.1.7 parental virus, consistent with a transmission advantage for B.1.1.7’s set of mutations.

## Highlights

• We find evidence for recombination in SARS-CoV-2
• We identify eight clear recombination events, four of which lead to onward transmission
• Estimated breakpoints are consistent with coronavirus cellular replication dynamics
• Transmitted recombinants inherited the more-transmissible B.1.1.7 spike gene

## Graphical abstract

**Figure:**
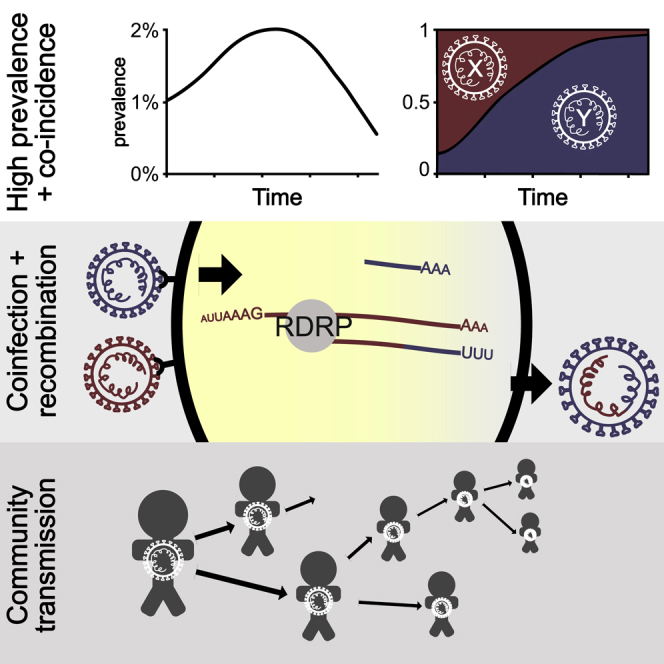

## Teaser

Sampling from late 2020 to early 2021 provides evidence for SARS-CoV-2 recombination and onward community transmission of recombinant viruses that inherited the spike region from the B.1.1.7 (alpha) variant.

## Introduction

Recombination, the transfer of genetic information between molecules derived from different organisms, is a fundamental process in evolution, because it can generate novel genetic variation upon which selection can act (Felsenstein, 1974). Genetic analysis indicates that recombination occurs frequently in betacoronaviruses (Lai et al., 1985; Keck et al., 1988; Lai and Cavanagh, 1997), including natural populations of MERS-CoV (Corman et al., 2014; Dudas and Rambaut, 2016; Kim et al., 2016) and SARS and SARS-like coronaviruses (Hon et al., 2008; Boni et al., 2020). The zoonotic transmission of an alphacoronavirus whose spike gene shows evidence of being the product of recombination between feline and canine coronaviruses has occurred in Malaysia, which demonstrates the potential for coronavirus recombination associated with host reservoirs (Vlasova et al., 2021). It has been proposed recently that the global SARS-CoV-2 genome sequence data contain signals of recombination across the pandemic (VanInsberghe et al., 2021). Recombination has the potential to be important in the context of pathogen evolution, because it can “rescue” genomes with otherwise deleterious mutations or provide the opportunity to create novel phenotypes by bringing genetic variation from different backgrounds onto a single genome. A concerning scenario from an epidemiological perspective is the potential for recombination to combine, in the same genome, mutations that may confer immune-escape properties with those that may enhance transmissibility. Enhanced transmissibility (Volz et al., 2021) and immune-escape (Planas et al., 2021) phenotypes have already been observed in SARS-CoV-2. Consequently, the characterization of recombination in SARS-CoV-2 is important for surveillance purposes.

The molecular mechanism of homologous recombination in unsegmented positive-sense RNA viruses such as SARS-CoV-2 is generally by copy-choice replication, a model first suggested in poliovirus (Copper et al., 1974). In this process, a hybrid or mosaic RNA is formed when the RNA-polymerase complex switches from one RNA template molecule to another during replication (Worobey and Holmes, 1999). In order for homologous recombination to occur and be subsequently detected, there must be coinfection of the same cell within an individual by genetically distinct viruses (termed the “parental” lineages of the recombinant virus). Coinfection of an individual requires there to be co-circulation of multiple viral lineages within a population and, given the short duration of most SARS-CoV-2 infections, is most likely to be observed when virus prevalence is high.

**Figure 1:**
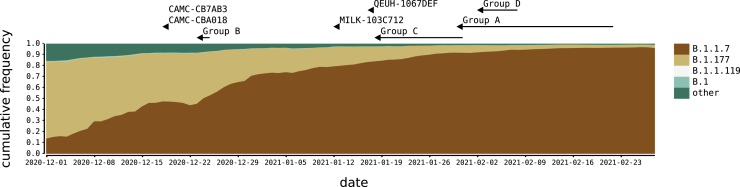
SARS-CoV-2 lineages in the UK, winter 2020–2021 The distribution of the most frequent SARS-CoV-2 lineages in the UK from December 2020 to February 2021. Here, B.1.177 refers to B.1.177, including all of its descendant lineages (e.g., B.1.177.9). For each recombinant or recombinant group, the date of the earliest sampled genome is indicated by an arrowhead. The recombination event that generated each must have occurred before this date. For groups A–D, the body of the arrow represents the range of dates that the samples span.

Conditions conducive to SARS-CoV-2 recombination existed in the United Kingdom (UK) during the latter part of 2020 and early in 2021. From mid-October 2020 to January 2021, SARS-CoV-2 prevalence was estimated to be between 1% and 2% in England (Steel and Hill, 2021). During this time, the B.1.1.7 variant of concern (VOC), which is also referred to as alpha under the World Health Organization nomenclature (WHO (World Health Organization), 2021), emerged, rapidly increased in frequency, and spread across the UK, replacing lineages that were already at high prevalence (Volz et al., 2021). The most common of the latter was the B.1.177 lineage and its descendants (Hodcroft et al., 2021) (Figure 1). B.1.1.7 is characterized by an unusually large number of genetic changes (22 mutations from its immediate ancestor; (Rambaut et al., 2020b)). The ability to detect virus recombination using comparative sequence analysis depends on the genetic distinctiveness of the parental viruses, so the co-circulation of B.1.1.7 and non-B.1.1.7 viruses is expected to increase the power to detect recombinants between these lineages. The UK’s high rate of genomic surveillance and unified collection of genomic, epidemiological, and geographic data also provide multiple lines of evidence for evaluating the identification of recombinant viruses.

To identify putative SARS-CoV-2 recombinant viruses, we carried out an analysis of all complete UK SARS-CoV-2 genomes that had been assigned to lineage B.1.1.7 and that showed evidence of being the product of combining different genetic lineages, indicative of recombination. Specifically, we scanned the UK dataset for genomes that were alternately composed of long contiguous tracts of B.1.1.7 and non-B.1.1.7 genetic variation. The genetic composition and epidemiological context of each candidate mosaic genome was carefully explored to determine whether it was recombinant in origin. We subsequently determined whether the recombinants showed evidence of onward transmission within the UK population. One recombinant lineage continued to circulate for at least 9 weeks and, as of May 5, 2021, was associated with 45 linked infections.

## Results

### Identification of putative recombinants

We identified a total of 16 recombinant sequences from the whole UK dataset of 279,000 sequences up to March 7, 2021 using our bioinformatic and evolutionary analysis pipeline (see STAR Methods). Twelve genome sequences that clustered into four groups (labeled A–D), and four additional singletons showed evidence of being mosaic in structure (Table 1; Table S1). For each group A–D, each of the constituent genomes was sampled from the same geographic locality within the UK (Table 1). For group A, which spanned two geographical regions, all the samples originated from close to the border between Wales and North West England (<20 km apart). The sample dates for the putative recombinants ranged from December 18, 2020 to February 2, 2021 (Figure 1). If groups A–D represent community transmission of a recombinant lineage with a single origin, recombination must have occurred on or before the date of the earliest sample in each group. It is possible to use molecular clock dating to infer bounds on recombination events, but given uncertainties in the rate of the molecular clock, estimates from such methods have large confidence intervals (Raghwani et al., 2012). Because our dataset consists of high-density genomic sampling covering the time period over which we infer recombination to have occurred, we use the earliest sample dates described above as representative of the approximate time of the recombination events. The range of dates coincides with a period of increasing relative prevalence of B.1.1.7 in the UK alongside the presence of other circulating lineages in the community, the most common of which were B.1.177 and its descendants (Figure 1).

**Table 1:** Recombinants and their putative second parental lineages according to genetic similarity

	No.	Location	Sample dates (year/month/day)	Second parent lineage	Second parent date (year/month/day)	Second parent location	Breakpoint coordinates
Group A	4	Wales/North West England	2021/01/30–2021/02/21	B.1.177	2021/01/27	Wales	21,255– 21765 (20,410–21,765)^a^
Group B	2	South East England	2020/12/23–2020/12/24	B.1.36.28	2020/12/10	Greater London	6,528–6,954
Group C	3	East Midlands (England)	2021/01/18–2021/01/30	B.1.221.1 / B.1.221.2	2021/01/02	East Midlands/West Midlands/Greater London	24,914–28,651
Group D	3	South East England	2021/02/02–2021/02/07	B.1.36.17	2020/12/10	East of England	21,575–23,063
CAMC-CBA018	1	Greater London	2020/12/18	B.1.177	2021/01/23	UK	11,396–21,991
CAMC-CB7AB3	1	Greater London	2020/12/18	B.1.177	2020/12/12	Greater London	3,267–6,286 and 12,534–21,765
MILK-103C712	1	Greater London	2021/01/12	B.1.177.16	2020/12/14	Greater London/East of England	26,801–27,972
QEUH-1067DEF	1	Scotland	2021/01/17	B.1.177.9	2021/01/13	Greater London	6,954–10,870

The first parental lineage is always B.1.1.7. For the recombinant groups, the number of genomes, the NUTS1 location of residence, and the range of sampling dates are given. Breakpoint coordinates are the range of possible SARS-CoV-2 genome positions bounded by mutations that are unambiguously inherited from one parent or the other, including both single-nucleotide polymorphisms and deletions. The date and location for the second parent is for the genetically most similar UK genome(s) within the genome region belonging to that lineage.

a For group A, the results for LIVE-DFCFFE (in parentheses) were different from those of the rest of the group.

**Figure S1:**
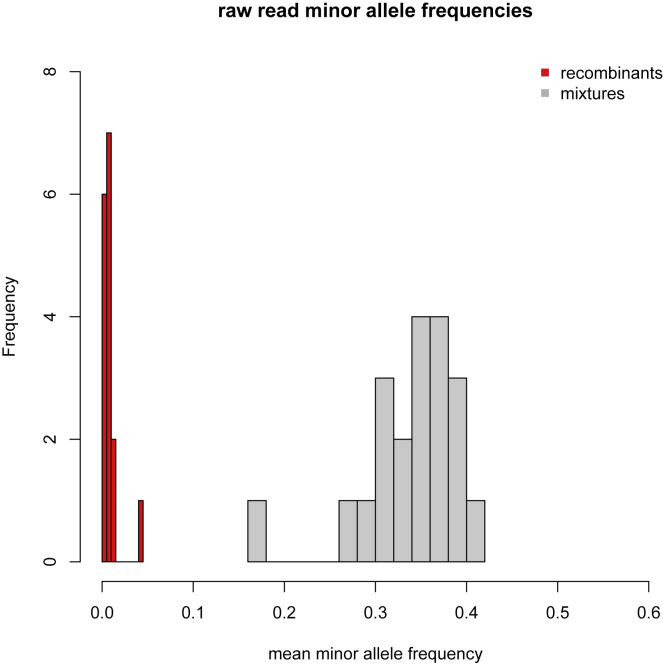
Read data minor allele frequencies, related to STAR Methods The distribution of minor allele frequencies from the read data for the 16 putative recombinants (red bars) and 20 samples suspected of being sequenced mixtures, either due to co-infection or laboratory contamination (gray bars). For each recombinant, the minor allele frequency is the mean across all sites that differ by a nucleotide change from the reference (MN908947.3) in it or either of its putative parentals by genetic similarity. For the mixtures, the minor allele frequency is the mean across the sites that differ by a nucleotide change from the reference at genomic positions where mutations occur in B.1.1.7.

To rule out the possibility that any of the 16 recombinants could have resulted from artifacts as a result of assembling sequence reads from a co-infected sample (generated through either natural coinfection or laboratory contamination), we examined the read coverage and minor allele frequencies and assessed the likelihood of a mixed sample. Several lines of evidence suggested the recombinant sequences were not the products of sequencing a mixture of genomes. First, the sequencing protocol used in the UK (Tyson et al., 2020) generates 98 short (∼350-bp) amplicons, such that long tracts that match just one lineage would be unlikely. Second, the read data do not support a mixture for any of the putative recombinant genomes. All the recombinants were sequenced to high coverage (lowest mean read depth per site per genome: 686; highest mean read depth: 2,903). The mean minor allele frequency (MAF) for the putative recombinants was 0.008, which is 6 standard deviations below the mean of the MAF (0.34) for a set of 20 sequences that we suspected to be mixtures (Figure S1). Finally, for all groups A–D, multiple genomes with the same mosaic structure were sequenced independently from different samples, and by different sequencing centers in the case of group A, implying that the original assembly was correct and, additionally, that transmission of the recombinant had occurred. All of the read data are available on the European Nucleotide Archive. Accession numbers are given in Table S2.

### Epidemiological information supports the identification of putative parental lineages

**Figure 2:**
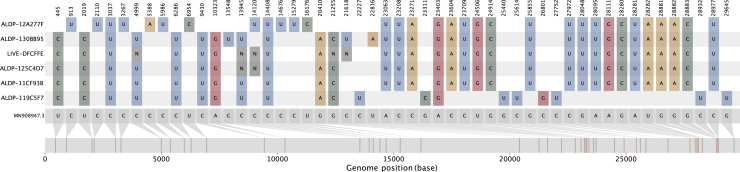
The nucleotide variation present in group A The nucleotide variation with respect to the reference sequence (MN908947.3; gray genome, far bottom) for the four members of group A (ALDP-11CF93B, ALDP-125C4D7, LIVE-DFCFFE, and ALDP-130BB95; middle four colored genomes) and their closest neighbors by genetic similarity among all UK sequences from the same time period for the B.1.1.7-like region of their genomes (ALPD-12A277F; top colored genome) and the B.1.177-like region of their genomes (ALDP-119C5F7; bottom colored genome). See also Figure S2.

**Figure S2:**
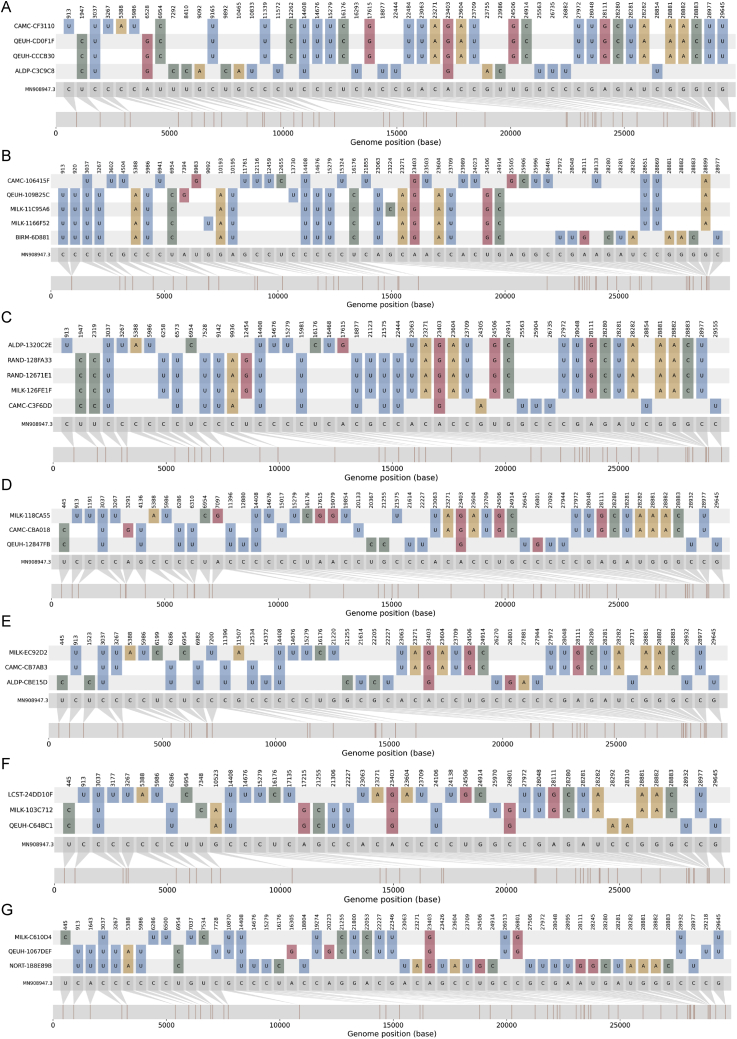
The nucleotide variation present in the recombinants and their parentals, related to Figure 2 The nucleotide variation with respect to the reference sequence (MN908947.3; gray genome far bottom) for each of the recombinant genomes (middle colored genomes in each panel) and their closest neighbors by genetic similarity among all UK sequences from the same time period, for the B.1.1.7-like and non-B.1.1.7-like regions of their genomes (top and bottom colored genomes in each panel). (A) Group B. (B) Group C. (C) Group D. (D) CAMC-CBA018. (E) CAMC-CB7AB3. (F) MILK-103C712. (G) QEUH-1067DEF.

**Figure S3:**
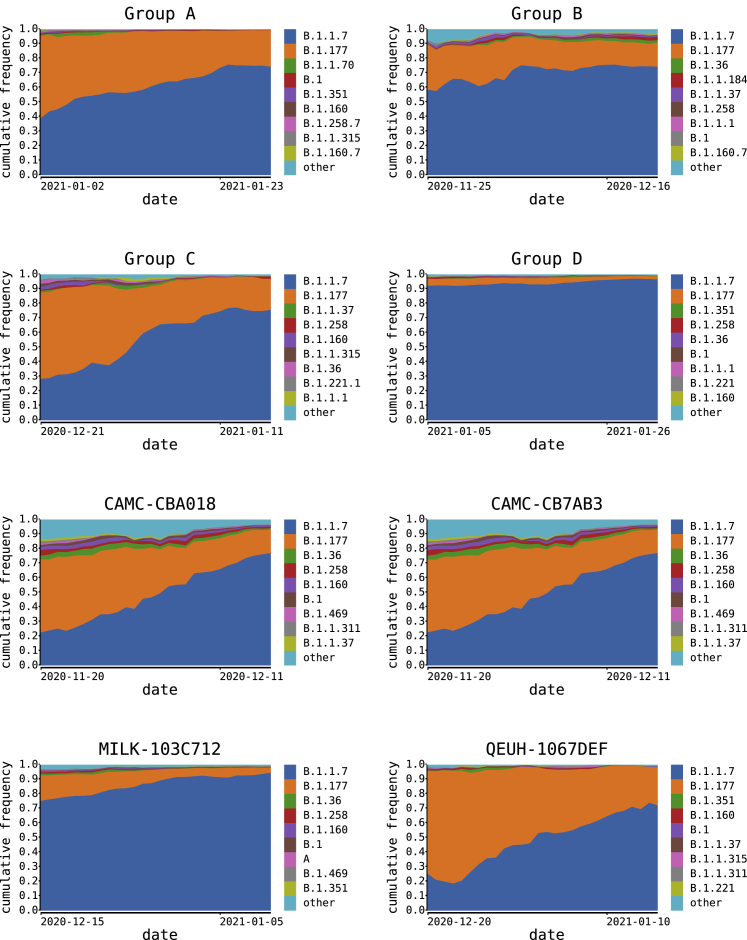
SARS-CoV-2 lineages in geographic regions of the UK relevant to the recombinants, related to Figure 1 The distribution of the most frequent SARS-CoV-2 lineages in the NUTS1 location of each set (Groups A-D and the four singletons) of recombinants for the four weeks immediately preceding each set’s (earliest) sample date. Here, B.1.177 refers to B.1.177 itself and all its descendant lineages (e.g., B.1.177.9); the same is true for B.1.36.

The nucleotide variation for the putative recombinants and their closest neighbors by genetic similarity (for each of the regions of their genomes either side of the recombination breakpoint) is shown in Figures 2 and S2. For each of groups A–D, the closest neighbors by genetic similarity for each of the two non-recombining genome regions were the same sequences for every putative recombinant within a group. For most of the recombinants, there were several equidistant putative parental sequences for each region of the genome; whenever this was true, they all belonged to the same lineage, except for group C, whose putative parental lineages for the non-B.1.1.7-like region of the genome were a mixture of two closely related lineages (B.1.221.1 and B.1.221.2). The putative parental sequence for the non-B.1.1.7 region of the genome varied by group (Table 1). Importantly, in each case, the sequence and epidemiological data demonstrate that the non-B.1.1.7 parental sequence was circulating in the same geographic area as the recombinant in the time immediately before the sampling date of the recombinant. For group A and the four singletons, the second parental sequence was assigned lineage B.1.177 or one of its descendants. B.1.177, which likely arose in Spain in the summer of 2020 and was exported to multiple European countries (Hodcroft et al., 2021), rose to high relative frequency in the UK through autumn 2020 and was widespread by December (Figure 1). Lineage B.1.177.16, the second parental sequence of MILK-103C712, was sampled 25 times in Greater London in the 4 weeks preceding MILK-103C712’s sample date. Lineage B.1.177.9 was sampled on one other occasion in Scotland in 4 weeks preceding QEUH-1067DEF’s sample date. Lineage B.1.36.28 was not sampled in South East England in the 4 weeks preceding group B’s sample date but was sampled eight times in Greater London in that period. Lineages B.1.221.1 and B.1.221.2 were sampled seven and zero times, respectively, in the East Midlands in the 4 weeks preceding group C’s sample date, and B.1.36.17 was sampled five times in the South East in the 4 weeks prior to group D’s sample date. The distributions of the most prevalent lineages in each region of the UK relevant to the eight sets of recombinants (groups A–D and the four singletons) for the 4 weeks immediately preceding the date of the earliest sampled genome in each case are shown in Figure S3.

### Putative recombinants exhibit significant mosaicism

We rejected the null hypothesis of non-reticulate evolution for 14 out of the 16 putative recombinant sequences by testing these sequences for mosaicism (3SEQ; Lam et al., 2018; with Dunn-Sidak correction for multiple comparisons) against a background set of 2,000 sequences randomly drawn from the course of the UK epidemic (Table 2). The lineages identified as the putative parentals assigned by 3SEQ agreed with the lineages for putative parentals assigned by genetic similarity (Tables 1 and 2), even though of the 16 closest neighbors by genetic similarity described above, none were present in the background sequence set of candidate parentals used in the 3SEQ analysis. The breakpoints reported by 3SEQ also agreed with breakpoints inferred from the distribution of single-nucleotide polymorphisms (SNPs) and deletions in the putative recombinants and their neighbors by genetic similarity (Tables 1 and 2). The two sequences that belong to group B did not show a statistically significant mosaic signal of non-reticulate evolution, but 3SEQ’s Δ_m,n,2_ statistic for these two candidate recombinants showed the greatest support for mosaicism possible among the ancestry-informative polymorphic sites with their closest neighbors by genetic similarity as parentals (n = 6, m = 42, k = 42). The associated uncorrected p value of 5.7e-7 does not survive a multiple comparisons correction due to the number of putative parental lineages and descendants that were tested (Table 2)

**Table 2:** Recombinants, their putative second parental lineages, and inferred breakpoints according to 3SEQ

	Second parent lineage	Uncorrected p value	Corrected p value	Breakpoint coordinates^a^
Group A	B.1.177	5E-13 (1E-12)^b^	0.00003 (0.00006)^b^	21,255–21,613 (18,998–20,294)^b^
Group B^c^		5.70E-07	1	6,528–6,953
Group C	B.1.221.1	4.26E-10	0.02686	25,996–27,441
Group D	B.1.36.39	3E-12	0.0002	20,703–23,062
CAMC-CBA018	B.1.177	2E-12	0.00014	20,389–21,254
CAMC-CB7AB3	B.1.177	1E-11	0.00065	3,267–4,474 and 20,389–21,254
MILK-103C712	B.1.177.17	1.06E-10	0.00673	408–444 and 26,801–27,876
QEUH-1067DEF	B.1.177.9	4.12E-10	0.026	10523 – 10869

The first parental lineage is always B.1.1.7. p values for the Δ_m,n,2_ statistic are reported uncorrected and after Dunn-Sidak correction for multiple comparisons.

a The breakpoint coordinates reported by 3SEQ are the positions of spaces between nucleotides.

b For group A, the results for LIVE-DFCFFE (in parentheses) were different from those of the rest of the group.

c Statistics are from a separate run with the closest neighbors by genetic similarity included in the parental dataset.

**Figure 3:**
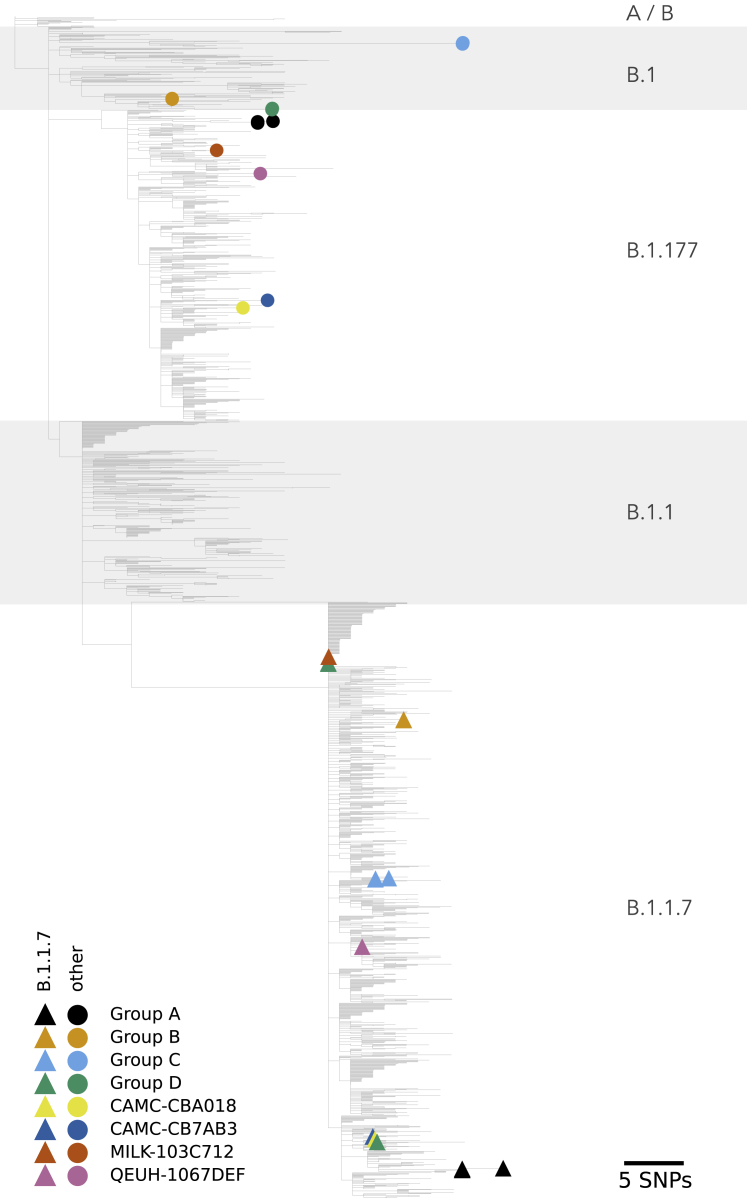
Phylogenetic placement of putative recombinant genome regions Phylogenetic reconstruction of 2,000 samples chosen to be representative of the course of the epidemic in the UK, as well as the 16 recombinant genomes, with their B.1.1.7-like part (colored triangles) and non-B.1.1.7-like part (colored circles) alternately unmasked. The tree is scaled by genetic divergence, and the scale in numbers of nucleotide changes is given in the bottom right of the Figure.

The Dunn-Sidak correction used in 3SEQ is very conservative, as it assumes that all 64.0 million comparisons we performed are independent statistical tests, when in fact these tests are highly nonindependent, since many candidate parental sequences are a small number of nucleotide differences apart from each other. When corrected p values are borderline, the recommended approach to infer reticulate evolution is to build separate phylogenetic trees for the nonrecombining regions of the genome to confirm that the recombinant in question has different phylogenetic placements in different genomic regions (Boni et al., 2010). With the exception of the inner region for CAMC-CB7AB3, whose placement within B.1.177 was not well supported, each recombinant’s two phylogenetic placements were with the lineages that we identified as parental by genetic similarity and by using 3SEQ, with high bootstrap support (Table S3). The placement of the two parental genome regions for each recombinant in the context of the whole epidemic in the UK is shown in Figure 3.

**Figure 4:**
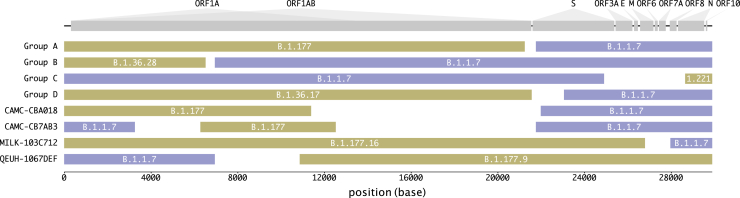
Mosaicism of putative recombinants Recombinant groups A–D contain multiple sequences exhibiting the same mosaic genome structures (see Table 1 for details). Tracts matching lineage B.1.1.7 are shown in blue, while virus genome regions matching other lineages are shown in yellow. Gaps represent ambiguity in the exact position of the recombinant breakpoints; there are no lineage-defining mutations within these regions. The breakpoint coordinates are taken from Table 1.

The mosaic structures of the genomes of the putative recombinants are shown in Figure 4. In six out of eight instances (and all four of the groups of >1 sequence, which may represent community transmission), the recombinants contain a spike gene from the B.1.1.7 lineage. In four instances, there is a proposed recombination breakpoint at or near the 5′ end of the spike gene.

### Further evidence for the community transmission of group A

**Figure 5:**
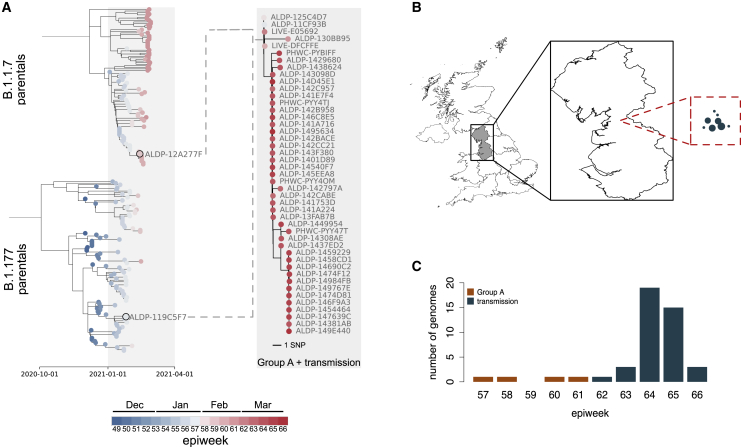
The community transmission of group A (A) The phylogenetic relationships between the closest genetic neighbors of group A for the B.1.1.7-inherited region of their genome (top clade; left-hand tree) and the B.1.177-inherited region of their genome (bottom clade; left-hand tree), with branch lengths scaled by time. The sample date in cumulative epidemiological weeks (epiweeks) since the first epiweek of 2020 for each sequence is also represented by colored circles at the tips of each tree; see the key for this scale. The closest two parental sequences by genetic similarity for the two regions of the genomes (ALDP-12A277F and ALDP-119C5F7) are labeled in the left-hand tree, and their tips are highlighted by black rings. The phylogenetic relationships within group A (top four taxa) and their descendants (bottom 41 taxa) are shown in the right-hand tree, with branch lengths scaled by divergence. The dashed lines represent the formation of a new recombinant clade between the members of group A and their parental lineages. (B) The geographic context of the transmitted recombinant sequences. The exploded region of the map is the North-West region of England. All of the 41 recombinants descended from group A were sampled in this region. The relative distribution of their locations, in the same scale as the exploded region, are represented by the circles in the red dashed square. The size of the points represents the number of genomes sequenced in each location. The absolute locations of the recombinants within North West England are not represented by this panel. (C) The distribution of the sampling dates for the 45 recombinants, aggregated by epiweek. Orange bars, four original members of group A; green bars, 41 descendants from group A.

**Figure S4:**
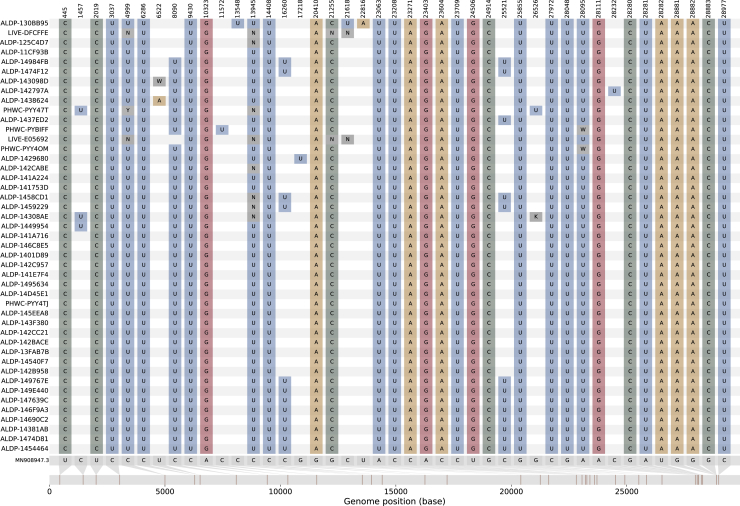
The nucleotide variation present in the descendants of group A, related to Figure 5 The distribution of nucleotide variation in the original members of group A (top four colored rows) and the 41 additional sequences that are derived from it (bottom 41 colored rows), with respect to the reference sequence (MN908947.3; very bottom gray sequence).

A follow-up investigation of the eight sets of putative recombinants (groups A–D and the four singletons) on May 5, 2021 found 41 sequences that were descended from group A (Figure 5). No descendants from any other recombinant event were detected to have continued to circulate. The 41 sequences share the same set of SNPs with three members of group A (ALDP-11CF93B, ALDP-125C4D7, and LIVE-DFCFFE), with additional nucleotide variation at positions 8,090, 16,260, and 25,521 (Figures 5A and S4). They were sampled in North West England between March 1, 2021 and April 4, 2021 (Figures 5B and 5C). The temporal distribution of samples descended from the recombination event that led to group A suggests that the recombinant lineage persisted at low frequency for a period of time before expanding and then contracting again (Figure 5C). A second follow-up investigation on July 14, 2021 found no further recombinants descended from group A, which suggests that this transmission cluster is extinct. The dynamics of this cluster of infections reflect the wider trend of SARS-CoV-2 prevalence in England over the same time period. Prevalence decreased from a maximum of 2.08% in January 2021 to 0.21% for the week beginning April 4, 2021 and to < 0.1% as of the beginning of May 2021 (Steel and Hill, 2021). Group A and its descendants met the criteria for designation as a recombinant Pango lineage (Pybus, 2021) and have been named lineage XA (https://cov-lineages.org/lineages/lineage_XA.html).

## Discussion

Here, we report the first unambiguous detection and characterization of the arisal and subsequent community transmission of recombinant SARS-CoV-2 viruses. Comparison of intra-genomic variation, supported by geographic and epidemiological data, demonstrates the occurrence of multiple independent recombination events involving UK virus lineages in late 2020. Recombinant genomes that share genetic identity were sampled from the same geographic location and time period, indicating they represent successful onward transmission after the occurrence of a single ancestral recombination event. In one instance, this resulted in a significant transmission cluster comprising 45 observed cases, which has been given the Pango lineage name XA. While no obvious biological advantage can be attributed to this cluster (or any of the observed recombinants) beyond the acquisition of B.1.1.7’s set of spike mutations, these recombinants are sentinel events for continued monitoring for new variants. With the increasing co-circulation of VOCs in the same geographic areas, careful monitoring is warranted.

Large-scale bioinformatic approaches have identified statistical signals of recombination among SARS-CoV-2 sequences using clade assignment and its changes along the genome as the primary characteristic under investigation (Varabyou et al., 2020; VanInsberghe et al., 2021). Due to the limited genetic diversity at the time these analyses were carried out, there was no strong statistical support for recombination (as opposed to non-reticulate diversification) for any particular candidate recombinant. When the number of mutations in a virus sequence is low (e.g., Figure 3 in VanInsberghe et al., 2021 and Figure 2 in Varabyou et al., 2020), there is generally little statistical support to reject the possibility that the sequence patterns could have been generated by mutation alone. In contrast, there is sufficient diversity in the UK virus dataset between the lineage B.1.1.7 and other co-circulating lineages to detect putative recombinants and demonstrate statistical significance for their breakpoint patterns. Candidate recombinants and their parental lineages are a median of 22.5-nt mutations apart, and candidate pairs of parentals are a median of 46-nt differences apart from each other (Figures 2 and S2). The p values we present are exact non-parametric probabilities that the observed patterns of nucleotide ancestry in the candidate recombinant viruses were generated by mutation alone given the parental genotypes and are corrected for multiple testing.

The breakpoint locations inferred from the recombinants’ parental lineages by two different methods and two different parental datasets are in agreement (cf. Tables 1 and 2). Two interesting observations arise from their distribution. First, in six cases, and in all the cases where we detected transmission, the spike gene was inherited from the B.1.1.7 parental sequence (Figure 4). This is consistent with the observed transmission advantage of B.1.1.7 (Volz et al., 2021), which is likely attributable to the mutations it carries in the spike region (Rambaut et al., 2020b). Second, in four instances, a breakpoint is located near the 5′ end of the spike gene. A defining feature of the order Nidovirales, to which coronaviruses belong, is the production during RNA synthesis of a set of nested positive- and negative-stranded subgenomic RNAs (sgRNAs) that contain a leader sequence derived from the 5′ end of the complete genome and a progressively reduced complement of the structural (S, E, M, N) and accessory genes, which form the body of the sgRNA molecule (Masters, 2006; Kim et al., 2020). The discontinuous nature of these sgRNAs is understood to be the product of template switching by viral polymerase during normal transcription, where the polymerase pauses at a transcription-regulatory sequence (TRS) after transcribing the last open reading frame (ORF) of the sgRNA and switches to a similar TRS upstream of the leader sequence (Sawicki and Sawicki, 1995), omitting a looped-out region of the template RNA, which contains at least orf1ab in the case of SARS-CoV-2 (Finkel et al., 2021). This provides an environment that is highly conducive to homologous recombination, including a polymerase that engages in template switching during its normal transcriptional activity and the availability of alternative template RNA molecules, in the form of sgRNAs, which incorporate sequence motifs that mediate template switching and might be derived from different genomes in the case of coinfection. As TRSs, which occur between the ORFs (Kim et al., 2020), are important in mediating template switching during homologous recombination in coronaviruses, this can account for the shared pattern of recombination-prone regions observed here (Figure 4). However, to be detected, recombinant genomes must lead to viable viruses, so the distribution of breakpoints observed from genomic surveillance may not represent the distribution of breakpoints that occur *in situ* (Banner and Lai, 1991).

Given the overall prevalence of SARS-CoV-2 in the UK in December 2020 of 1% (Steel and Hill, 2021) and assuming that 40% of infections were of lineage B.1.1.7 and 40% of B.1.177 (Figure 1), a simplistic expectation for the number of coinfections involving these two lineages is the product of their prevalences, which is 16 coinfections per million people. The figure is 70 coinfections per million people for a prevalence of 2%, 70% infection with B.1.1.7, and 25% infection with B.1.177, which applies to the first half of January 2021. In this calculation, we assume that infections are independent and that once infected, the chance of an additional infection occurring is unchanged. This illustrates that on a national scale, coinfection should be quite common during periods of high prevalence. From a public health perspective, this reminds us that halving prevalence reduces the chance of coinfection by a factor of four, because the probability of coinfection increases with the square of the prevalence.

As recombination permits the combination of advantageous mutations from distinct variants and recombination is only possible with coinfection, minimizing the prevalence of SARS-CoV-2 will minimize the chance of forming recombinant lineages with genetic combinations that could potentially increase virus fitness. At the time of writing, the trajectory of the SARS-CoV-2 pandemic continues to remind us that there are many populations worldwide still highly susceptible to large epidemic waves. High-prevalence epidemic waves comprising diverse viral lineages risk high rates of meaningful recombination, and as SARS-CoV-2 genetic diversity is much greater in 2021 than in 2020, it will be important to examine each epidemic for the presence of novel recombinant lineages, especially epidemics occurring in regions with circulation of different variants of biological significance.

### Limitations of study

In the Results, we discuss the possibility that the mosaic patterns of genetic variation observed in the putative recombinant genome sequences might be the product of some process other than recombination, for example the sequencing of a natural coinfection or of a mixed sample due to laboratory contamination. However, neither the distribution of the raw read allele frequencies for the putative recombinants (which are unlike those we observe in samples that we suspect to be real mixtures) nor the spatial distribution of genetic variation along their genomes (consisting of long contiguous tracts compatible with a single lineage, which is not the pattern expected for a mixture of samples given the sequencing protocol employed in the UK) suggests that this is the case. In the case of the groups A–D, the sequencing of multiple distinct samples with nearly identical genetic variation provides strong *a posteriori* evidence against any of these genomes being the product of sequencing a mixed sample. This strength of evidence does not exist for the four singleton genomes, so they should be viewed as less certainly recombinant.

## STAR★Methods

### Key resources table

**Table:** 

REAGENT or RESOURCE	SOURCE	IDENTIFIER
**Deposited data**		

Virus genome sequencing data	European Nucleotide Archive	See Table S2

**Software and algorithms**		

Grapevine	Github repository	https://github.com/COG-UK/datapipe
type_variants	Github repository	https://github.com/cov-ert/type_variants
gofasta	Github repository	https://github.com/cov-ert/gofasta
snipit	Github repository	https://github.com/aineniamh/snipit
Sequencing coverage	Volz et al. (2021)	https://github.com/robj411/sequencing_coverage
Samtools	Li et al. (2009)	https://github.com/samtools/samtools
3SEQ	Lam et al. (2018)	https://mol.ax/software/3seq/
IQtree	Minh et al. (2020)	http://www.iqtree.org/
TreeTime	Sagulenko et al. (2018)	https://github.com/neherlab/treetime

### Resource availability

#### Lead contact

Further information and requests for resources and reagents should be directed to and will be fulfilled by the Lead Contact, Ben.Jackson@ed.ac.uk.

#### Materials availability

This study did not generate new unique reagents.

### Method details

#### Identification of putative recombinants

A national SARS-CoV-2 sequencing effort in the UK, the COG-UK consortium (COVID-19 Genomics UK (COG-UK (COVID-19 Genomics UK), 2020), has undertaken systematic genomic surveillance of SARS-CoV-2 in the country and generated over 440,000 genomes to date. As part of the COG-UK daily analytical pipeline (https://github.com/COG-UK/datapipe), the consensus genome sequences of the complete set of UK samples were aligned to the SARS-CoV-2 reference sequence (GenBank: MN908947.3) using Minimap2 (Li, 2018). The aligned sequences were converted from sam to fasta format, and each assigned a Pango lineage (Rambaut et al., 2020a) using Pangolin (O’Toole et al., 2021). Pango lineages are designed to capture ongoing epidemiological trends at a resolution suitable for genomic epidemiology and outbreak investigation (Rambaut et al., 2020a). From the sequence alignment, we extracted all sequences that had been assigned to lineage B.1.1.7, up to the 2021/03/07. We genotyped these sequences at the set of 22 sites that discriminate B.1.1.7 from its parental lineage (B.1.1) using a custom script in Python (https://github.com/cov-ert/type_variants), then discarded sequences with missing data at any of the 22 sites. We visualized the resulting table of genotype calls in order to identify sequences that showed evidence of a potential mosaic genome structure (i.e., runs of contiguous sites that were not compatible with the B.1.1.7 lineage designation).

#### Identification of candidate parental sequences

To identify candidate parental genome sequences in a computationally-tractable manner we created a set of all UK SARS-CoV-2 sequences that (i) contained no N nucleotide ambiguity codes after masking the 3′ and 5′ UTRs, (ii) spanned the dates 2020/12/01 to 2021/02/28, which represents two weeks before the date of the earliest putative recombinant, to one week after the date of the latest, and (iii) excluded the putative recombinant genomes identified above. This set consisted of 98859 sequences in total. For each putative recombinant, we split its genome sequence into B.1.1.7-like regions and non-B.1.1.7 regions at the junction of genetic regions according to the mosaic structure detected by the custom Python script described above (https://github.com/cov-ert/type_variants; Table S1). Then for each component region of each mosaic genome, we first masked the remainder of the genome with Ns (in both the focal mosaic sequence and all background sequences) then found the most-genetically similar non-focal sequences by computing pairwise genetic distances (number of nucleotide differences per site) using gofasta (https://github.com/cov-ert/gofasta). Subsequently, an alignment was compiled for each putative recombinant, which contained the putative recombinant as well as the most-genetically similar background sequences (as identified above) for each component region of that mosaic genome. The single nucleotide differences between the putative recombinant and the closely related reference sequences were visualized using snipit (https://github.com/aineniamh/snipit). The genomic coordinates of the boundaries between each mosaic genome region were then refined by taking into account observed lineage-defining nucleotide and deletion variation. Specifically, we set the boundary coordinates to the ends of sequential tracts of mutations specific to the putative parental sequences. This is a conservative approach to assigning parental lineages and consequently no parental lineage is assigned to those genome regions that do not contain unambiguous lineage-defining mutations or deletions. Lastly, using these refined region boundaries, we reiterated the genetic distance calculation above to identify a final set of most-genetically similar sequences for each putative recombinant.

When reporting geographic locations for UK virus genome sequences we use level 1 of the Nomenclature of Territorial Units for Statistics (NUTS) geocode standard (https://ec.europa.eu/eurostat/web/nuts/history).

#### Defining a representative sample from the UK epidemic

To generate a limited set of genomes that are suitable for computationally-expensive analysis yet are also representative of the genetic diversity of the SARS-CoV-2 epidemic in the UK, we randomly sampled 2000 sequences from 21st March 2020, when sequence data first became available, to 1st March 2021, weighting the probability of choosing a sequence accounting for the sequencing coverage and covid19 prevalence in individual geographic regions of the UK over time, using the same method as in Volz et al. (2021), which is available at (https://github.com/robj411/sequencing_coverage). We use this dataset to investigate the phylogenetic placement of the alternate regions of recombinant genomes, and as a dataset of putative parental sequences to statistically test for recombination using 3SEQ.

#### Investigation of read data

Almost all sequencing sites in the COG-UK consortium use the ARTIC PCR protocol to produce tiled PCR amplicons, which are then sequenced (Tyson et al., 2020). The generated sequence reads are then processed using sequence mapping, rather than sequence assembly, to produce a consensus genome for each sample. This approach, which was designed to support epidemiological investigations, creates a single consensus sequence for each sample. Beyond representing sites with high minor allele frequencies using the appropriate IUPAC nucleotide alphabet ambiguity code, this consensus does not reflect the natural genetic variation of SARS-CoV-2 genomes observed within an infected individual (Lythgoe et al., 2021). Mapping is particularly suited to tiled amplicons generated from samples that contain limited genomic diversity. Further, mapping is typically less prone to introducing errors/artifacts than sequence assembly and enables effective primer sequence removal and identification of non-reference mutations. Genomic sites that exhibit intra-sample nucleotide variation could be consistent with a range of processes, including co-infection, within-patient diversity, contamination, or PCR error. The identification of such sites forms part of the consensus-generating pipeline, and we exploit that information here in order to rule out the possibility that our mosaic consensus sequence represents a mixture of virus genomes, rather than representing true recombinant genomes.

For each putative recombinant sequence we analyzed the original read data from virus genome sequencing in order to rule out the possibility that the generated consensus sequence represents a mixture of virus genomes (due to laboratory contamination or coinfection, for example), rather than representing a true recombinant genome. To do this we calculated minor allele frequencies (MAFs) from the read data and compared their distribution between the 16 recombinant genomes and 20 samples that we suspected of being the product of sequencing a mixture of genomes, potentially because of coinfection or laboratory contamination. To define sequences that we suspected of being mixtures, we scanned the dataset for consensus sequences that possessed an IUPAC ambiguity code at the 27 genomic positions that differ from the SARS-CoV-2 reference genome (GenBank: MN908947.3) by a nucleotide change in B.1.1.7 (the 27 positions include those with nucleotide changes that were inherited from the ancestor of B.1.1.7). We define the MAF at a single site as the number of sequencing reads not containing the most frequently observed single nucleotide allele that mapped to that site, divided by the total number of sequencing reads that include any nucleotide allele that mapped to that site. For each virus genome, we defined a set of genomic positions from which to calculate MAF as follows. For each recombinant, we considered every site that differed from MN908947.3 by a nucleotide in its own consensus genome, or in the consensus genome of either of its parentals by genetic similarity. For the sequences that we suspected of being mixtures we considered the 27 genomic positions where sequences belonging to B.1.1.7 differ from MN908947.3 by a nucleotide change. We used samtools (Li et al., 2009), with default filters for mapping and base quality, to extract allele calls from the read data using its mpileup subroutine, and to calculate mean read depth per genome using its depth subroutine.

### Quantification and statistical analysis

#### Test for mosaic genome structure

We used 3SEQ (Lam et al., 2018) as a statistical test for recombination in the UK SARS-CoV-2 data. 3SEQ interrogates triplets of sequences for a signal of mosaicism in one “child” sequence, given the genotypes of the other two “parental” sequences, using an exact non-parametric test for clustering in a sequence of binary outcomes (Boni et al., 2007). The test statistic Δ_m,n,2_ used in 3SEQ simply tests if a putative recombinant’s ancestry in parental A clusters in the middle of the genome, while ancestry in parental B clusters in the outer regions of the genome. We manually adjusted two-breakpoint recombinants to be single-breakpoint recombinants if one of the breakpoints according to 3SEQ abutted the beginning or end of the genome. We tested all potential pairs of sequences from the representative parental dataset from the course of the UK pandemic (n = 2000) against each putative recombinant in the child dataset (n = 16), and report p values that are uncorrected and that are Dunn-Sidak corrected for multiple comparisons (n = 64.0 million). We performed a single additional run of 3SEQ with two putative recombinant sequences that were not found to be significantly the mosaic product of any of the sequences in the representative background as children, and their closest neighbors by genetic similarity as parentals. P values for this test were reported without correction and after correction for multiple testing assuming that this test was in addition to the 64 million comparisons that we had already performed. The input and output files for the 3SEQ analysis are available at https://github.com/COG-UK/UK-recombination-analysis.

#### Test for the phylogenetic incongruence of putative recombinant genome tracts

For each of the eight sets of recombinants (Groups A-D and the four singletons) we carried out the following procedure to test for incongruence between the phylogenetic placements of the two regions of their genomes. We independently added each set’s genome(s) to the representative background of 2000 sequences, along with the reference sequence, to create eight alignments in total. We masked the resulting alignments according to the breakpoints defined by the closest neighbors by genetic similarity, so that for each set, we produced two sub-alignments: one consisting of the region that was inherited from the B.1.1.7 parental in the recombinant(s), and one consisting of the region that was inherited from the other parental. This resulted in 16 alignments in total. We reconstructed the phylogenetic relationships for each with IQTREE v2.1 (Minh et al., 2020), using the HKY model of nucleotide substitution, conducting 1000 ultrafast bootstrap replicates (Minh et al., 2013; Hoang et al., 2018), and rooting the tree on the reference sequence, which is basal to all B lineage sequences. The phylogenetic trees produced by this analysis are available at https://github.com/COG-UK/UK-recombination-analysis.

To determine the placement of the different regions of each recombinant genome in a single context, we also built a phylogenetic tree of the representative background’s complete genomes, to which we added the masked recombinant genomes, so that each recombinant was present in the alignment twice, once with the B.1.1.7 region of its genome unmasked, and once with the opposing region unmasked. We ran IQTREE as above.

#### Follow up of putative recombinants

To test for onward community transmission of the putative recombinants, we searched the whole UK dataset as of the 5th May 2021 for additional sequences whose genetic variation matched the variation of the recombinants. For each of the eight set of recombinants, we defined a set of SNPs and deletions by which all the recombinants within that set differed from the reference sequence (MN908947.3). Then we used type_variants to scan the UK dataset for genomes whose SNP and deletion variation was compatible with being a descendant or sibling of the putative recombinants. Group A represented the only recombination event with evidence for further transmission according to the results of this procedure. We carried out the following additional analyses to further investigate transmission of Group A genomes. First, we visualized the nucleotide variation of the additional matching genomes using snipit and extracted their sampling locations and dates. Second, to explore the phylogenetic context of Group A and its derivatives, we reconstructed their (whole-genome) phylogenetic relationships using IQTREE. We also extracted the 100 closest sequences by genetic similarity for each alternate region of the genome (B.1.1.7-like and non-B.1.1.7-like) for each of the four original members of Group A to provide phylogenetic context to the parental sequences. This resulted in a dataset of 216 sequences in total when the two groups of neighbors were combined, and duplicates removed. We reconstructed their (whole-genome) phylogenetic relationships with the IQTREE, as above. We generated a time-scaled phylogenetic tree from the divergence tree of parental sequences using TreeTime (Sagulenko et al., 2018), setting the–clock-rate parameter to 0.001. We labeled the phylogenetic tree of recombinants and the phylogenetic tree of parental sequences with the sampling date in number of epidemiological weeks (epiweeks) since the first epiweek of 2020 to assess the temporal context of the recombination event and subsequent transmission. We carried out a second follow up on 14^th^ July 2021 using the same procedure as above.

## Consortia

The members of the COG-UK Consortium are Samuel C. Robson, Tanya Golubchi, Rocio T. Martinez Nunez, Catherine Ludden, Sally Corden, Ian Johnston, David Bonsall, Colin P. Smith, Ali R. Awan, Giselda Bucca, M. Estee Torok, Kordo Saeed, Jacqui A. Prieto, David K. Jackson, William L. Hamilton, Luke B. Snell, Catherine Moore, Ewan M. Harrison, Sonia Goncalves, Leigh M. Jackson, Ian G. Goodfellow, Derek J. Fairley, Matthew W. Loose, Joanne Watkins, Rich Livett, Samuel Moses, Roberto Amato, Darren L. Smith, Jeff Barrett, David M. Aanensen, Martin D. Curran, Surendra Parmar, Dinesh Aggarwal, James G. Shepherd, Matthew D. Parker, Sharon Glaysher, Matthew Bashton, Anthony P. Underwood, Katie F. Loveson, Alessandro M. Carabelli, Kate E. Templeton, Cordelia F. Langford, John Sillitoe, Thushan I. de Silva, Dennis Wang, Dominic Kwiatkowski, Justin O’Grady, Simon Cottrell, Matthew T.G. Holden, Emma C. Thomson, Husam Osman, Monique Andersson, Anoop J. Chauhan, Mohammed O. Hassan-Ibrahim, Mara Lawniczak, Ravi Kumar Gupta, Alex Alderton, Meera Chand, Chrystala Constantinidou, Meera Unnikrishnan, Julian A. Hiscox, Steve Paterson, Inigo Martincorena, Erik M. Volz, Andrew J. Page, Andrew R. Bassett, Cristina V. Ariani, Michael H. Spencer Chapman, Kathy K. Li, Rajiv N. Shah, Natasha G. Jesudason, Yusri Taha, Martin P. McHugh, Rebecca Dewar, Aminu S. Jahun, Claire McMurray, Sarojini Pandey, James P. McKenna, Andrew Nelson, Gregory R. Young, Clare M. McCann, Scott Elliott, Hannah Lowe, Ben Temperton, Sunando Roy, Anna Price, Sara Rey, Matthew Wyles, Stefan Rooke, Sharif Shaaban, Mariateresa de Cesare, Laura Letchford, Siona Silveira, Emanuela Pelosi, Eleri Wilson-Davies, Myra Hosmillo, Andrew R. Hesketh, Richard Stark, Louis du Plessis, Chris Ruis, Helen Adams, Yann Bourgeois, Stephen L. Michell, Dimitris Grammatopoulos, Jonathan Edgewort, Judith Breuer, John A. Todd, Christophe Fraser, David Buck, Michaela John, Gemma L. Kay, Steve Palmer, Sharon J. Peacock, David Heyburn, Danni Weldon, Esther Robinson, Alan McNally, Peter Muir, Ian B. Vipond, John Boyes, Venkat Sivaprakasam, Tranprit Salluja, Samir Dervisevic, Emma J. Meader, Naomi R. Park, Karen Oliver, Aaron R. Jeffries, Sascha Ott, Ana da Silva Filipe, David A. Simpson, Chris Williams, Jane A.H. Masoli, Bridget A. Knight, Christopher R. Jones, Cherian Koshy, Amy Ash, Anna Casey, Andrew Bosworth, Liz Ratcliffe, Li Xu-McCrae, Hannah M. Pymont, Stephanie Hutchings, Lisa Berry, Katie Jones, Fenella Halstead, Thomas Davis, Christopher Holmes, Miren Iturriza-Gomara, Paul Anthony Randell, Alison Cox, Pinglawathee Madona, Kathryn Ann Harris, Julianne Rose Brown, Tabitha W Mahungu, Dianne Irish-Tavares, Tanzina Haque, Jennifer Hart, Eric Witele, Melisa Louise Fenton, Steven Liggett, Clive Graham, Emma Swindells, Jennifer Collins, Gary Eltringham, Sharon Campbell, Patrick C. McClure, Gemma Clark, Tim J. Sloan, Carl Jones, Jessica Lynch, Ben Warne, Steven Leonard, Jillian Durham, Thomas Williams, Nathaniel Storey, Nabil-Fareed Alikhan, Nadine Holmes, Christopher Moore, Matthew Carlile, Malorie Perry, Noel Craine, Ronan A. Lyons, Angela H. Beckett, Salman Goudarzi, Christopher Fearn, Kate Cook, Hannah Dent, Hannah Paul, Robert Davies, Beth Blane, Sophia T. Girgis, Mathew A. Beale, Katherine L. Bellis, Matthew J. Dorman, Eleanor Drury, Leanne Kane, Sally Kay, Samantha McGuigan, Rachel Nelson, Liam Prestwood, Shavanthi Rajatileka, Rahul Batra, Rachel J. Williams, Mark Kristiansen, Angie Green, Anita Justice, Adhyana I.K. Mahanama, Buddhini Samaraweera, Nazreen F. Hadjirin, Joshua Quick, Leanne M. Kermack, Nicola Reynolds, Grant Hall, Yasmin Chaudhry, Malte L. Pinckert, Iliana Georgana, Robin J. Moll, Alicia Thornton, Richard Myers, Joanne Stockton, Charlotte A. Williams, Wen C. Yew, Alexander J. Trotter, Amy Trebes, George MacIntyre-Cockett, Alec Birchley, Alexander Adams, Amy Plimmer, Bree Gatica-Wilcox, Caoimhe McKerr, Ember Hilvers, Hannah Jones, Hibo Asad, Jason Coombes, Johnathan M. Evans, Laia Fina, Lauren Gilbert, Lee Graham, Michelle Cronin, Sara Kumziene-SummerhaYes, Sarah Taylo, Sophie Jones, Danielle C. Groves, Peijun Zhang, Marta Gallis, Stavroula F. Louka, Igor Starinskij, Chris J. Illingworth, Chris Jackson, Marina Gourtovaia, Gerry Tonkin-Hill, Kevin Lewis, Jaime M. Tovar-Corona, Keith James, Laura Baxter, Mohammad T. Alam, Richard J. Orton, Joseph Hughes, Sreenu Vattipally, Manon Ragonnet-Cronin, Fabricia F. Nascimento, David Jorgensen, Olivia Boyd, Lily Geidelberg, Alex E. Zarebski, Jayna Raghwani, Moritz U.G. Kraemer, Joel Southgate, Benjamin B. Lindsey, Timothy M. Freeman, Jon-Paul Keatley, Joshua B. Singer, Leonardo de Oliveira Martins, Corin A. Yeats, Khalil Abudahab, Ben E.W. Taylor, Mirko Menegazzo, John Danesh, Wendy Hogsden, Sahar Eldirdiri, Anita Kenyon, Jenifer Mason, Trevor I. Robinson, Alison Holmes, James Price, John A. Hartley, Tanya Curran, Alison E. Mather, Giri Shankar, Rachel Jones, Robin Howe, Sian Morgan, Elizabeth Wastenge, Michael R. Chapman, Siddharth Mookerjee, Rachael Stanley, Wendy Smith, Timothy Peto, David Eyre, Derrick Crook, Gabrielle Vernet, Christine Kitchen, Huw Gulliver, Ian Merrick, Martyn Guest, Robert Munn, Declan T. Bradley, Tim Wyatt, Charlotte Beaver, Luke Foulser, Sophie Palmer, Carol M. Churcher, Ellena Brooks, Kim S. Smith, Katerina Galai, Georgina M. McManus, Frances Bolt, Francesc Coll, Lizzie Meadows, Stephen W. Attwood, Alisha Davies, Elen De Lacy, Fatima Downing, Sue Edwards, Garry P. Scarlett, Sarah Jeremiah, Nikki Smith, Danielle Leek, Sushmita Sridhar, Sally Forrest, Claire Cormie, Harmeet K. Gill, Joana Dias, Ellen E. Higginson, Mailis Maes, Jamie Young, Michelle Wantoch, Sanger Covid Team (https://www.sanger.ac.uk/covid-team), Dorota Jamrozy, Stephanie Lo, Minal Patel, Claire M. Bewshea, Sian Ellard, Cressida Auckland, Ian Harrison, Chloe Bishop, Vicki Chalker, Alex Richter, Andrew Beggs, Angus Best, Benita Percival, Jeremy Mirza, Oliver Megram, Megan Mayhew, Liam Crawford, Fiona Ashcroft, Emma Moles-Garcia, Nicola Cumley, Richard Hopes, Patawee Asamaphan, Marc O. Niebel, Rory N. Gunson, Amanda Bradley, Alasdair Maclean, Guy Mollett, Rachel Blacow, Paul Bird, Thomas Helmer, Karlie Fallon, Julian Tang, Antony D. Hale, Louissa R. Macfarlane-Smith, Katherine L. Harper, Holli Carden, Nicholas W. Machin, Kathryn A. Jackson, Shazaad S.Y. Ahmad, Ryan P. George, Lance Turtle, Elaine O’Toole, Joanne Watts, Cassie Breen, Angela Cowell, Adela Alcolea-Medina, Themoula Charalampous, Amita Patel, Lisa J. Levett, Judith Heaney, Aileen Rowan, Graham P. Taylor, Divya Shah, Laura Atkinson, Jack C.D. Lee, Adam P. Westhorpe, Riaz Jannoo, Helen L. Lowe, Angeliki Karamani, Leah Ensell, Wendy Chatterton, Monika Pusok, Ashok Dadrah, Amanda Symmonds, Graciela Sluga, Zoltan Molnar, Paul Baker, Stephen Bonner, Sarah Essex, Edward Barton, Debra Padgett, Garren Scott, Jane Greenaway, Brendan A.I. Payne, Shirelle Burton-Fanning, Sheila Waugh, Veena Raviprakash, Nicola Sheriff, Victoria Blakey, Lesley-Anne Williams, Jonathan Moore, Susanne Stonehouse, Louise Smith, Rose K. Davidson, Luke Bedford, Lindsay Coupland, Victoria Wright, Joseph G. Chappell, Theocharis Tsoleridis, Jonathan Ball, Manjinder Khakh, Vicki M. Fleming, Michelle M. Lister, Hannah C. Howson-Wells, Louise Berry, Tim Boswell, Amelia Joseph, Iona Willingham, Nichola Duckworth, Sarah Walsh, Emma Wise, Nathan Moore, Matilde Mori, Nick Cortes, Stephen Kidd, Rebecca Williams, Laura Gifford, Kelly Bicknell, Sarah Wyllie, Allyson Lloyd, Robert Impey, Cassandra S. Malone, Benjamin J. Cogger, Nick Levene, Lynn Monaghan, Alexander J. Keeley, David G. Partridge, Mohammad Raza, Cariad Evans, Kate Johnson, Emma Betteridge, Ben W. Farr, Scott Goodwin, Michael A. Quail, Carol Scott, Lesley Shirley, Scott A.J. Thurston, Diana Rajan, Iraad F. Bronner, Louise Aigrain, Nicholas M. Redshaw, Stefanie V Lensing, Shane McCarthy, Alex Makunin, Carlos E. Balcazar, Michael D. Gallagher, Kathleen A. Williamson, Thomas D. Stanton, Michelle L. Michelsen, Joanna Warwick-Dugdale, Robin Manley, Audrey Farbos, James W. Harrison, Christine M. Sambles, David J. Studholme, Angie Lackenby, Tamyo Mbisa, Steven Platt, Shahjahan Miah, David Bibby, Carmen Manso, Jonathan Hubb, Gavin Dabrera, Mary Ramsay, Daniel Bradshaw, Ulf Schaefer, Natalie Groves, Eileen Gallagher, David Lee, David William, Nicholas Ellaby, Hassan Hartman, Nikos Manesis, Vineet Patel, Juan Ledesma, Katherine A. Twohig, Elias Allara, Clare Pearson, Jeffrey K.J. Cheng, Hannah E. Bridgewater, Lucy R. Frost, Grace Taylor-Joyce, Paul E. Brown, Lily Tong, Alice Broos, Daniel Mair, Jenna Nichols, Stephen N. Carmichael, Katherine L. Smollett, Kyriaki Nomikou, Elihu Aranday-Cortes, Natasha Johnson, Seema Nickbakhsh, Edith E. Vamos, Margaret Hughes, Lucille Rainbow, Richard Eccles, Charlotte Nelson, Richard Gregory, Matthew Gemmell, Chloe L. Fisher, Adrian W. Signell, Gilberto Betancor, Harry D. Wilson, Gaia Nebbia, Flavia Flaviani, Alberto C. Cerda, Tammy V. Merrill, Rebekah E. Wilson, Marius Cotic, Nadua Bayzid, Thomas Thompson, Erwan Acheson, Steven Rushton, Sarah O’Brien, David J. Baker, Steven Rudder, Alp Aydin, Fei Sang, Johnny Debebe, Sarah Francois, Tetyana I. Vasylyeva, Marina Escalera Zamudio, Bernardo Gutierrez, Angela Marchbank, Joshua Maksimovic, Karla Spellman, Kathryn McCluggage, Mari Morgan, Robert Beer, Safiah Afifi, Trudy Workman, William Fuller, Catherine Bresner, Adrienn Angyal, Luke R. Green, Paul J. Parsons, Rachel M. Tucker, Rebecca Brown, Max Whiteley, James Bonfield, Christoph Puethe, Andrew Whitwham, Jennifier Liddle, Will Rowe, Igor Siveroni, Thanh Le-Viet, Amy Gaskin, and Rob Johnson.

## Data Availability

All the code used to perform the analyses here is available at: https://github.com/COG-UK/UK-recombination-analysis
