# Supplementary material for: Generation and transmission of interlineage recombinants in the SARS-CoV-2 pandemic
Source: Cell. 2021 Sep 30;184(20):5179–5188.e8. doi: 10.1016/j.cell.2021.08.014 (PMC8367733; doi:10.1016/j.cell.2021.08.014)
Supplement: Document S1. Tables S1–S3 [file mmc1.pdf]

Cell, Volume *184*

Supplemental information

Generation and transmission of interlineage recombinants in the SARS-CoV-2 pandemic

Ben Jackson, Maciej F. Boni, Matthew J. Bull, Amy Colleran, Rachel M. Colquhoun, Alistair C. Darby, Sam Haldenby, Verity Hill, Anita Lucaci, John T. McCrone, Samuel M. Nicholls, Áine O'Toole, Nicole Pacchiarini, Radoslaw Poplawski, Emily Scher, Flora Todd, Hermione J. Webster, Mark Whitehead, Claudia Wierzbicki, The COVID-19 Genomics UK (COG-UK) Consortium, Nicholas J. Loman, Thomas R. Connor, David L. Robertson, Oliver G. Pybus, and Andrew Rambaut

Table S1. The first iteration of mosaic genome structure detection for the putative recombinants. (See STAR Methods; see also Tables 1 and 2).

Nucleotide, amino acid, or deletion states at the 22 sites where mutations define lineage B.1.1.7 from its immediate ancestor, for each putative recombinant. The genotype column names are in one of the following formats: (i) “snp”, followed by the nucleotide in MN908947.3 (reference), the position in the MN908947.3 genome, then the nucleotide in B.1.1.7; (ii) “aa” (amino acid), followed by the name of the coding sequence, the residue in MN908947.3, the position of the residue in the coding sequence, then the residue in B.1.1.7; (iii) “del” (deletion) followed by the first position of the deletion, then the length of the deletion. For the contents of the amino acid columns, an asterisk (*) denotes a stop codon. For the contents of the deletion genotype columns, del=deletion present; ref=deletion absent. Lineage B.1.1.7 states are coloured red, and MN908947.3 alleles are coloured white. The mosaic structures visible here informed the first iteration of recombination detection.

Sample Name	Group	Sample Date	snp:C913T	aa:ORF1ab:T1001I	aa:ORF1ab:A1708D	snp:C5986T	aa:ORF1ab:I2230T	del:11288:9	snp:C14676T	snp:C15279T	snp:T16176C	del:21765:6	del:21991:3	aa:S:N501Y	aa:S:A570D	aa:S:P681H	aa:S:T716I	aa:S:S982A	aa:S:D1118H	aa:ORF8:Q27*	aa:ORF8:R52I	aa:ORF8:Y73C	aa:N:D3L	aa:N:S235F
CAMC-CBA018	-	18/12/2020	C	T	A	C	I	ref	C	C	T	del	del	Y	D	H	I	A	H	*	I	C	L	F
ALDP-11CF93B	A	30/01/2021	C	T	A	C	I	ref	C	C	T	del	del	Y	D	H	I	A	H	*	I	C	L	F
ALDP-125C4D7	A	06/02/2021	C	T	A	C	I	ref	C	C	T	del	del	Y	D	H	I	A	H	*	I	C	L	F
LIVE-DFCFFE	A	14/02/2021	C	T	A	C	I	ref	C	C	T	del	del	Y	D	H	I	A	H	*	I	C	L	F
ALDP-130BB95	A	21/02/2021	C	T	A	C	I	ref	C	C	T	del	del	Y	D	H	I	A	H	*	I	C	L	F
QEUH-CCCB30	B	23/12/2020	C	T	A	C	T	del	T	T	C	del	del	Y	D	H	I	A	H	*	I	C	L	F
QEUH-CD0F1F	B	24/12/2020	C	T	A	C	T	del	T	T	C	del	del	Y	D	H	I	A	H	*	I	C	L	F
MILK-1166F52	C	24/01/2021	T	I	D	T	T	del	T	T	C	del	del	Y	D	H	I	A	H	Q	R	Y	D	S
MILK-11C95A6	C	30/01/2021	T	I	D	T	T	del	T	T	C	del	del	Y	D	H	I	A	H	Q	R	Y	D	S
QEUH-109B25C	C	18/01/2021	T	I	D	T	T	del	T	T	C	del	del	Y	D	H	I	A	H	Q	R	Y	D	S
MILK-126FE1F	D	07/02/2021	C	T	A	C	I	ref	C	C	T	ref	ref	Y	D	H	I	A	H	*	I	C	L	F
RAND-12671E1	D	02/02/2021	C	T	A	C	I	ref	C	C	T	ref	ref	Y	D	H	I	A	H	*	I	C	L	F
RAND-128FA33	D	02/02/2021	C	T	A	C	I	ref	C	C	T	ref	ref	Y	D	H	I	A	H	*	I	C	L	F
CAMC-CB7AB3	-	18/12/2020	T	I	A	C	I	ref	C	C	T	del	del	Y	D	H	I	A	H	*	I	C	L	F
MILK-103C712	-	12/01/2021	C	T	A	C	I	ref	C	C	T	ref	ref	N	A	P	T	S	D	*	I	C	L	F
QEUH-1067DEF	-	17/01/2021	T	I	D	T	T	ref	C	C	T	ref	ref	N	A	P	T	S	D	Q	R	Y	D	S

Table S2. GISAID and ENA accession numbers for the 16 putative recombinant sequences. (See STAR Methods).

Sample	Group	GISAID accession	ENA sample accession	ENA sample secondary accession	ENA run accession
ALDP-11CF93B	A	EPI_ISL_989697	ERS5764793	SAMEA8077663	ERR5308556
ALDP-125C4D7	A	EPI_ISL_1019487	ERS5792357	SAMEA8105282	ERR5323237
ALDP-130BB95	A	EPI_ISL_1122630	ERS5883487	SAMEA8196877	ERR5414941
LIVE-DFCFFE	A	EPI_ISL_1104468	ERS5872630		ERR5404883
QEUH-CCCB30	B	EPI_ISL_782203	ERS5522602	SAMEA7775362	ERR5058070
QEUH-CD0F1F	B	EPI_ISL_782442	ERS5522673	SAMEA7775433	ERR5058141
MILK-1166F52	C	EPI_ISL_938901	ERS5697106	SAMEA8009867	ERR5272107
MILK-11C95A6	C	EPI_ISL_1050799	ERS5874115		ERR5406307
QEUH-109B25C	C	EPI_ISL_909004	ERS5669824	SAMEA7982538	ERR5232711
MILK-126FE1F	D	EPI_ISL_1057512	ERS5812369	SAMEA8125397	ERR5349458
RAND-12671E1	D	EPI_ISL_1045772	ERS5805519	SAMEA8118538	ERR5335088
RAND-128FA33	D	EPI_ISL_1042110	ERS5809401	SAMEA8122425	ERR5340986
CAMC-CBA018	-	EPI_ISL_777766	ERS5517714	SAMEA7770468	ERR5054123
CAMC-CB7AB3	-	EPI_ISL_777974	ERS5517673	SAMEA7770427	ERR5054082
MILK-103C712	-	EPI_ISL_994038	ERS5760589	SAMEA8073454	ERR5304348
QEUH-1067DEF	-	EPI_ISL_917983	ERS5674718	SAMEA7987439	ERR5238288

Table S3. Phylogenetic placement and UF bootstrap support for the two parental tracts of each recombinant's genome, among the whole UK epidemic. (See STAR Methods).

For CAMC-CB7AB3, "Left" refers to the inner part of its genome, and "Right" refers to the outer part of its genome. The full phylogenies with bootstrap support are available at https://github.com/COG-UK/UK-recombination-analysis

Sample	Group	Left placement	Left support	Right placement	Right support
ALDP-11CF93B	A	B.1.177	81	B.1.1.7	100
ALDP-125C4D7	A	B.1.177	81	B.1.1.7	100
LIVE-DFCFFE	A	B.1.177	81	B.1.1.7	100
ALDP-130BB95	A	B.1.177	81	B.1.1.7	100
QEUH-CCCB30	B	B.1.36.28	97	B.1.1.7	100
QEUH-CD0F1F	B	B.1.36.28	97	B.1.1.7	100
MILK-1166F52	C	B.1.1.7	100	B.1.221.1	98
MILK-11C95A6	C	B.1.1.7	100	B.1.221.1	98
QEUH-109B25C	C	B.1.1.7	100	B.1.221.1	98
MILK-126FE1F	D	B.1.36.17/B.1.36.39	100	B.1.1.7	100
RAND-12671E1	D	B.1.36.17/B.1.36.39	100	B.1.1.7	100
RAND-128FA33	D	B.1.36.17/B.1.36.39	100	B.1.1.7	100
CAMC-CB7AB3	-	B.1.177	39	B.1.1.7	100
CAMC-CBA018	-	B.1.177	94	B.1.1.7	100
MILK-103C712	-	B.1.177	100	B.1.1.7	100
QEUH-1067DEF	-	B.1.1.7	100	B.1.177	100
